# Influence of the Processing Parameters on the Fiber-Matrix-Interphase in Short Glass Fiber-Reinforced Thermoplastics

**DOI:** 10.3390/polym9060221

**Published:** 2017-06-13

**Authors:** Anna Katharina Sambale, Marc Schöneich, Markus Stommel

**Affiliations:** Chair of Plastics Technology, TU Dortmund University, Leonard-Euler Str. 5, D-44227 Dortmund, Germany; marc.schoeneich@tu-dortmund.de (M.Sc.); markus.stommel@tu-dortmund.de (M.St.)

**Keywords:** polymer-matrix composites, interphase, nano-scratch, mechanical testing, injection molding, short glass fiber composites

## Abstract

The interphase in short fiber thermoplastic composites is defined as a three-dimensional, several hundred nanometers-wide boundary region at the interface of fibers and the polymer matrix, exhibiting altered mechanical properties. This region is of key importance in the context of fiber-matrix adhesion and the associated mechanical strength of the composite material. An interphase formation is caused by morphological, as well as thermomechanical processes during cooling of the plastic melt close to the glass fibers. In this study, significant injection molding processing parameters are varied in order to investigate the influence on the formation of an interphase and the resulting mechanical properties of the composite. The geometry of the interphase is determined using nano-tribological techniques. In addition, the influence of the glass fiber sizing on the geometry of the interphase is examined. Tensile tests are used in order to determine the resulting mechanical properties of the produced short fiber composites. It is shown that the interphase width depends on the processing conditions and can be linked to the mechanical properties of the short fiber composite.

## 1. Introduction

Plastics are versatile materials that have gained an increasingly important role as a structural and functional material in recent decades. A significant increase in the strength and stiffness of plastics is achieved by introducing reinforcing particles into the polymer matrix [1]. Especially short glass fiber-reinforced thermoplastics are widely used in several industrial applications to create high performance, lightweight parts and devices. The combination of stiff glass fibers in a thermoplastic matrix yields an anisotropic composite material with improved mechanical properties especially in the direction of the fiber orientation [1]. The mechanical properties of the composite are largely dependent on the local microstructure and the interaction between the different material phases. Particularly the adhesion between the fibers and the matrix is crucial to ensure an optimum load transfer from the matrix phase into the glass fibers in order to improve the macroscopic composite performance [2,3].

At the microscale, recent literature demonstrates the presence of a third phase between short glass fibers and the polymer matrix [4]. Generally, this interphase region is defined as a zone at the interface of the matrix and the fiber exhibiting modified polymer properties [5]. Thus, this region represents an additional, three-dimensional material phase [6,7] whereupon the differing mechanical properties compared to the bulk material are induced by morphological and thermomechanical effects [5,8,9].

In semi-crystalline composites, the fiber-matrix interphase varies from several hundred nanometers to a few microns in width around the glass fiber [5,10]. Particularly, the fiber-matrix adhesion and thus the interphase are affected by the choice of glass fiber sizing and the conditions of crystallization during the cooling of the melt [11,12,13]. While cooling down, the fibers are acting as nucleation agents leading to a high density of spherulitic structures around the fiber, which is one explanation for the existence of an interphase. If the crystalline structures are growing mainly perpendicular to the fiber surface, the created morphology is described as trans-crystallinity [14]. The formation of columnar structures on the fiber surface, for example row-nucleated cylindritic structures, is possible due to melt shearing. The appearance and the difference between trans-crystallinity and shear-induced crystalline structures are studied by Varga and Karger-Kocsis [15].

Further studies regarding the effects of trans-crystallization and their impact on the interphase region are given by Quan et al. and Bergeret et al. [16,17]. 

Since crystalline structures are linked to the presence of an interphase, the conditions for the crystallization process greatly influence the formation of the interphase region. These process conditions include the rate of cooling, the crystallization temperature and the prevailing pressure during crystallization [16]. During the injection molding process for the manufacturing of short fiber plastic parts, different process parameter settings determine the cooling of the melt. The resulting cooling conditions alter the crystallization process and thus the morphology of the polymer matrix linked to the mechanical composite properties [11].

The formation of an interphase region is also dependent on the surface texture, the roughness and the surface energy of the glass fibers and the corresponding sizing [17,18].

The relation between the fiber sizing and the interphase is studied by Mäder [19] for semi-crystalline thermoplastics. The glass fiber sizing represents a thin layer applied to the fibers during the manufacturing process, which protects the fibers from mechanical surface damage [20]. In addition, the sizing contributes strongly to the fiber-matrix adhesion by maximizing the shear strength at the interface between the glass fiber and the polymer matrix by means of a chemical affinity. To ensure a good fiber-matrix adhesion, an organosilicon compound is typically used as a component of the sizing [3]. These silane compounds are acting as adhesion promoters and are constituted by the chemical structure $R-SiX_{3}$ whereby *X* is a hydrolyzable group and *R* refers to an organic radical [3]. The silane adhesion promoter is bound to the glass fiber surface through hydrogen bonding and covalent siloxane bonds, whereas the polymer matrix chains are linked to the reactive radical of the silane [21]. A further prerequisite for good fiber-matrix adhesion is a complete wetting of the sizing on the glass fibers [22]. In order to improve the adhesion, the sizing not only has to be adapted to the glass fibers, but also to the surrounding polymer matrix. However, the composition of an adhesive promoter providing an optimized adhesion to the polymer matrix is still a challenging issue [18].

In contrast to semi-crystalline plastics, amorphous thermoplastics do not contain molecular chains forming crystalline structures. However, an interphase can still be expected based on the interdiffusion of macromolecules between the matrix material and the glass fiber sizing controlled by thermodynamic forces [23]. The exact mechanisms in terms of the interdiffusion and the resulting adhesion of the fibers with the matrix have still not been fully understood [9,10,13,24].

In the literature, different experimental methods for the geometrical mapping and mechanical characterization of the interphase are discussed. Friedrich et al. provide transmission electron microscopy (TEM) images of the crystalline regions around fibers. Here, trans-crystalline structures of thin layers in microfibrillar reinforced composites are studied by TEM. Thomason et al. show transgranular zones due to isothermal crystallization around glass fibers by means of polarized light microscopy. Varga et al. identify different shear-induced crystalline structures by pulling embedded fibers in the quiescent melt on a hot stage, imaging the crystalline structures by using a polarizing optical microscope [15]. The mechanical properties of the interphase are determined by Cech et al. [5] using atomic force microscopy (AFM). For this purpose, the surface of the glass fiber-reinforced samples is scanned in the tapping mode of the AFM to determine the surface topography, as well as the phase shift. However, the measurements using AFM only provide near-surface information about the interphase, which is highly dependent on the sample preparation [25]. For a mechanical characterization of the interphase region, AFM and nano-indentation are applied in order to examine local changes regarding contact stiffness and microhardness [8,9,11].

The local interphase width between the fiber and the matrix phase can be measured by means of nano-scratching. For this purpose, the sample is placed in contact with the nano-indenter tip and is moved at a constant speed. Consequently, the tip scratches successively across each phase (polymer matrix, interphase, glass fiber). Following a measurement method developed by Schöneich et al. [4], the normal force is determined in order to maintain a constant scratch depth between the matrix and the glass fiber. By analyzing the measured normal force over the scratch path, the interphase geometry within the cutting plane of the sample can be identified [4].

The present work is focused on the influences concerning the formation of a fiber-matrix interphase in short fiber-reinforced thermoplastics. In this study, the thermoplastic polybutylene terephthalate (PBT) is used as the matrix material. The glass fiber sizing and the process parameters of the specimen production are considered to have an impact on the formation of an interphase. Therefore, a systematic study is conducted. The measurement of the interphase is performed by means of nano-scratches in accordance with the method of Schöneich et al. [4]. Four hypotheses on the formation of the interphase are proposed as a part of this study:

**Hypothesis** **1.** The fiber sizing affects the fiber-matrix adhesion and the fracture properties due to the formation of an interphase:

The influence of the glass fiber sizing on the formation of an interphase is investigated by means of two compounded composite granulates. Coated and uncoated glass fibers are compounded in a PBT matrix using a twin screw extruder. Tensile specimens, consisting of the compounded granulates, are produced via the injection molding process. The processing parameters for the granulate with sized fibers and for the granulate with unsized fibers are equal and adjusted following the product data sheets. For the characterization of the macroscopic composite properties, tensile tests are carried out. For the measurement of the interphase width, nano-scratches are performed using parts taken from the tensile specimen. Subsequently, the resulting mechanical properties are correlated with the available microscale interphase information.

**Hypothesis** **2.** The interphase formation and its width are influenced by the processing parameters of the injection molding process through crystallization kinetics:

Regarding the processing effects on the interphase, the present study investigates different injection molding parameters using the statistical tool “design of experiments (DoE)”. For this purpose, the parameters “melt temperature”, “mold temperature” and “holding pressure” are varied during the processing of different samples. The parameters “melt temperature” and “mold temperature” set the kinetics of the solidification and are related to the crystallization behavior of the polymer matrix [26]. The holding-pressure is considered, since the injection molding process causes a volume contraction during solidification of the melt resulting in a shrinkage of the final part. In order to compensate component shrinkage during the holding pressure phase, an additional melt cushion is used after the complete filling of the cavity. The involved compression affects the crystallization behavior during the cooling phase, since the kinetics of crystallization from the melt change under different pressure conditions [16]. Consequently, the adjustment of the holding pressure, i.e., the amount of compression of the melt, is suspected to influence the interphase formation.

**Hypothesis** **3.** The interphase is not only a crystallization phenomenon for semi-crystalline composites:

In order to exclude the effects of semi-crystalline thermoplastics on the fiber-matrix interphase, additional samples are made from amorphous thermoplastics. In this case, acrylonitrile butadiene styrene (ABS) specimens are fabricated to study the existence of the interphase in comparison to previous semi-crystalline samples.

**Hypothesis** **4.** The width of the interphase correlates with the mechanical behavior of thermoplastics:

In addition to the measurement of the width of the interphase by nano-scratches, the mechanical properties of the different injection molded samples are determined via tensile tests with load directions parallel to the fiber orientation, as well as perpendicular to the fiber orientation. The width of the interphase and the mechanical properties’ tensile strength, the stiffness and the elongation at break are compared in order to determine a possible correlation.

## 2. Materials

A polybutylene terephthalate reinforced with 20 wt % of short glass fibers (PBT GF20) is used. The applied “Pocan^®^ B 3225” is a commercial-grade material, which is provided by the company Lanxess (Cologne, Germany). The PBT matrix of the composite is a semi-crystalline thermoplastic and the composite is used in a wide range of technical applications [27]. The relevant material properties are listed in Table 1 [28]. Detailed information about the glass fiber sizing is not provided by the manufacturer.

In addition to the semi-crystalline PBT-GF20, an amorphous acrylonitrile butadiene styrene (ABS) reinforced with 20 wt % glass fibers (“Polyman FABS-20-GF”) from the company A. Schulman (Akron, OH, USA) is investigated. The material properties of Polyman FABS-20-GF are illustrated in Table 2 [29]. There are no specifications given about the used glass fibers and the glass fiber sizing in the composite.

Furthermore, sized and unsized glass fibers are compounded in a PBT matrix material using a twin screw extruder Thermo Fisher Scientific, Waltham, MA, USA). The PBT matrix material used for this purpose is provided by the company Lanxess (Cologne, Germany). The material properties of the PBT matrix material (“Pocan^®^ B 1305”) can be found in Table 3 [30].

A composite granulate with sized glass fibers “GF CS 7968” (chopped strands) and a composite granulate with unsized glass fibers “GF MF 7980” (milled fibers) from Lanxess are compounded. The material properties of the two glass fiber types are shown in Table 4 [31,32].

## 3. Experimental Methods

Different specimen types are manufactured in order to determine the manifold influences on the interphase formation. The procedure is subdivided into in three steps: the composite production, the processing of the specimen and an experimental analysis. The specimen processing and sample analysis distinguish between the following influences on the interphase:

● Influence of the glass fiber sizing:

To produce a short glass fiber-reinforced composite, sized glass fibers (GF CS 7968) and unsized glass fibers (GF MF 7980) are added to a pure PBT matrix material (Pocan B 1305 from Lanxess) via compounding using a twin screw extruder. The compounded granulate types are injection molded involving the same processing parameters from a reference specimen type produced with an industrial-grade PBT-GF20 material. The influence of the glass fiber sizing on the formation of an interphase is determined by a comparison to the reference specimens.

● Influence of the processing parameters of the injection molding:

By using design of experiments (DoE), the influences of different injection molding parameters “melt temperature”, “mold temperature” and “holding pressure” on the interphase formation are determined. A reference specimen type is processed according to the product data sheet. Based on these reference injection molding parameters, various specimen types are produced by varying the three factors according to an experimental design. The used granulate type for the specimens of the experimental design is a PBT-GF20 Pocan B 3225 of the company Lanxess. The effects of the injection molding factors are identified via mechanical testing and nano-scratches.

● Influence of the matrix morphology:

To identify the influence of the semi-crystalline matrix material PBT, an amorphous thermoplastic matrix (ABS) reinforced with 20 wt % of glass fibers (ABS-GF20 from A. Schulman) is also used to fabricate specimens. The injection molding parameters are chosen according to product data sheets. A determination of the interphase is carried out to identify the interphase thickness.

### 3.1. Composite Production

Short glass fiber-reinforced thermoplastics can be processed continuously by extrusion or discontinuously by injection molding in the melting phase. Both processing operations are used in this study to produce specimens from the required materials. At first, the compounding of the two different composite types, consisting of PBT matrix material and sized fibers for the composite, as well as unsized glass fibers are considered. These composites are used to identify the influence of a glass fiber sizing on the interphase. The compounding of the two granulate types is processed by means of a co-rotating twin screw extruder.

The used Thermo Fisher Scientific “Haake OS Rheomex PTW16” twin screw extruder is composed of two modular constructed screws in the inside of a barrel, parted into ten heating zones. Two different granulates are produced by compounding. At first, the PBT-matrix material and sized glass fibers (BC) are compounded, and the output composite strand is cut to granulate pieces by using a fly cutter. A second granulate type is manufactured the same way by compounding unsized glass fibers combined with the PBT matrix material (UC). The fiber content of the composite is set to a weight ratio of 8:2 to compare the compounded granulates with the industrial processed ones. The process parameters are kept constant during the compounding of the two granulate types. The compounding parameters are listed in Table 5.

### 3.2. Specimen Processing

The different specimen types and the utilized materials for the specimen production are listed in Table 6. The different specimen are fabricated with the help of the injection molding machine “Arburg Allrounder 370 S 700-290 U” (Arburg, Loßburg, Germany).

In pilot tests, the optimal parameter settings are determined with the help of the utilized granulate, the given specimen geometry and their dimensional accuracy, as well as the product data sheets of the materials. The listed processing parameters of the injection molding in Table 7 are kept constant for all injection molded specimens.

These determined parameters are defined to be the reference parameters. In further steps, the barrel temperature, the holding pressure level, as well as the mold temperature are varied in order to investigate the influence of the processing parameters regarding the interphase formation. In Table 7, the represented parameters are varied in the context of an experimental design based on the parameters for the PBT-GF20 reference specimens, whereas the processing parameters to manufacture ABS-GF20 specimens are kept constant.

### 3.3. Design of Experiments

The influence of the injection molding parameters on the interphase formation in short glass fiber-reinforced thermoplastics is studied by applying a statistical experimental design.

Fractional factorial experimental designs are applied to reduce time-consuming pretests. For this study, a screening design with a resolution step of III is chosen. This design determines the influences of three factors in four tests.

The parameters “melt temperature”, “holding pressure” and “mold temperature” are considered to be the crucial influencing factors on the crystallization. In pilot tests, specimens are produced according to the product data sheet, and the determined injection molding parameters are designated as reference settings.

Based on the reference settings, low (“−1”) and high (“+1”) levels for the three varying factors “mass temperature”, “level of holding pressure” and “mold temperature” are chosen at a constant distance from the reference setting. The remaining injection molding parameters are kept constant during the entire test duration. The chosen levels are at equal distance from the reference parameters. Additional pilot tests verify the system to be executable between the chosen levels. Table 8 shows the high, the low and the reference level settings for the three factors, respectively.

By using the software MiniTab 17 (MiniTab Inc., State College, PA, USA), a statistical program for the evaluation of experiments, a fractional factorial design involving the level settings from Table 8 is generated and listed in Table 9.

**Figure 1:**
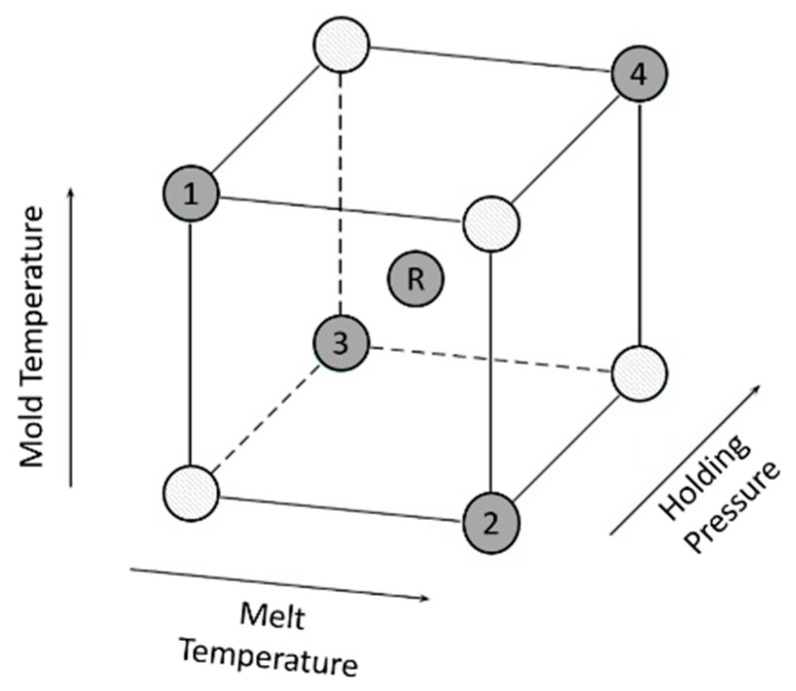
Applied settings of the experimental space for the fractional factorial design after [33].

The first column of Table 9 defines the succession of each test. The following columns specify the level settings of the three factors “melt temperature in (°C)”, “holding pressure in (bar)” and “mold temperature in (°C)”. Figure 1 shows the experimental space for the applied fractional factorial design. The darker spheres in the experimental space mark the performed factor settings of the design.

After a resetting of the injection molding parameters, the first three processed specimens are rejected for every setting in order to obtain constant conditions while processing. Additionally, attention was paid to a low residence time of the melt within the heated plasticizing unit to avoid thermal degradation of the melt [34].

### 3.4. Specimen Preparation and Testing

A specimen preparation is performed for the analysis after the processing procedure. To prepare the specimens for the different testing methods, they are detached from their sprue system. To determine the width of the interphase, the examination area is extracted by cutting the specimen. The extracted sample is ground and polished in serval steps.

The determination of the interphase is performed by a nano-scratch method. This method is used to measure the width of the interphase in the composite material. Table 10 lists the applied parameters for the nano-scratch tests.

For the characterization of the stress-strain behavior of the respective material, all tensile tests are performed at a constant strain rate of 1 mm/min. The internal pressure tests perpendicular to the fiber orientation in the specimen proceed, including with 2 cm^3^/min of water, which refers to a strain rate of 1.3%/min in the peripheral direction. In order to obtain reliable average properties, five repetitions of the tests are performed. A digital image correlation (DIC) camera system of the company LIMESS and the analysis program ISTRA 4D (Limess, Krefeld, Germany) are used to measure the strain during the performed tests.

The wall thicknesses of the tube specimens are measured by a magnetostatic sensor called MiniTest 7200 FH 4 of the company Elektrophysik (Cologne, Germany). The applied parameters of the tensile tests are shown in Table 11.

## 4. Determination of the Interphase

**Figure 2:**
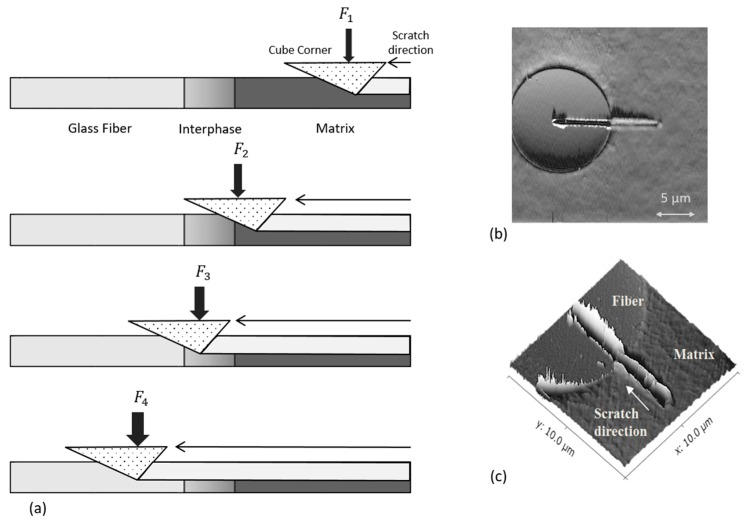
Nano-scratch with constant scratch depth following [35]: (**a**) representation of the scratch path at constant depth from the matrix to the fiber; (**b**) imaging of the PBT-GF20 sample after nano-scratching; (**c**) 3D-imaging of (**b**).

The local interphase width can be measured by means of nano-scratching, as shown in Figure 2. In this case, the sample is moved at a constant speed in contact with the nano-indenter tip such that the tip scratches from the matrix over the interphase to the fiber. In the present study, the local interphase width is measured by means of nano-scratching with a Hysitron^®^ TI 900 TriboIndenter (Bruker Hysitron, Minneapolis, USA).

The methodology developed by Schöneich et al. [4] is applied. During the nano-scratch, the sample is moved at a constant speed of 0.1 µm/s while the contact with the nano-indenter tip is maintained. As shown in Figure 2a, the penetration depth of the tip is kept constant by the normal force being controlled during the process.

In order to avoid artefacts from the sample surface topography, a pre-scratch is first performed. For that purpose, the tip is initially immersed 100 nm into the sample. Thereafter, the main scratch proceeds along an identical scratch path with 150 nm of penetration depth to the surface. The uniformity of the material pile-up displays the advantage of this approach. In addition, the nano-indenter tip is continuously located at the same depth in the material. Thus, the engaged contact area of the indenter tip and the sample is constant, whereby no correction factor is required to calculate the self-imaging effect of the tip.

Figure 2b,c displays the nano-scratch paths according to the described methodology. In order to determine the interphase width, different slopes are identified through the measured normal force progression during nano-scratching. 

**Figure 3:**
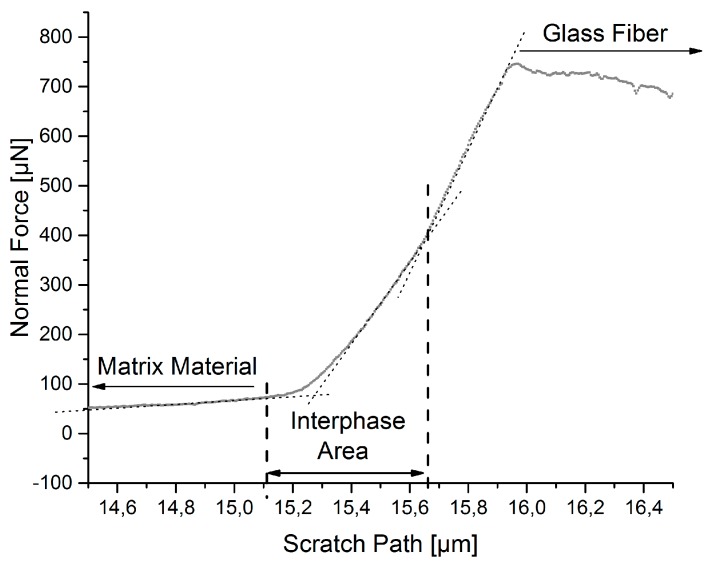
Measured normal force over the scratch path, identification of the interphase width using the slope method.

Figure 3 shows a representative normal force profile of a nano-scratch coming from the matrix to the glass fiber. A nearly constant normal force is measured over the scratch path in the area of the pure matrix and fiber material. Due to the presence of different slopes between the matrix and fiber phases, the width of the interphase area can be identified. The onset of the interphase starts with the deviation from the linear gradient from the matrix material. The subsequent linear increase in the normal force refers to the interphase. This interphase area ends with a further increase of the normal force. The renewed rise of the normal force is referred to the initial contact of the indenter tip with the pure fiber material. Further information about the measurement of the interphase width by nano-scratches can be taken from the studies of Schöneich et al. [4].

**Figure 4:**
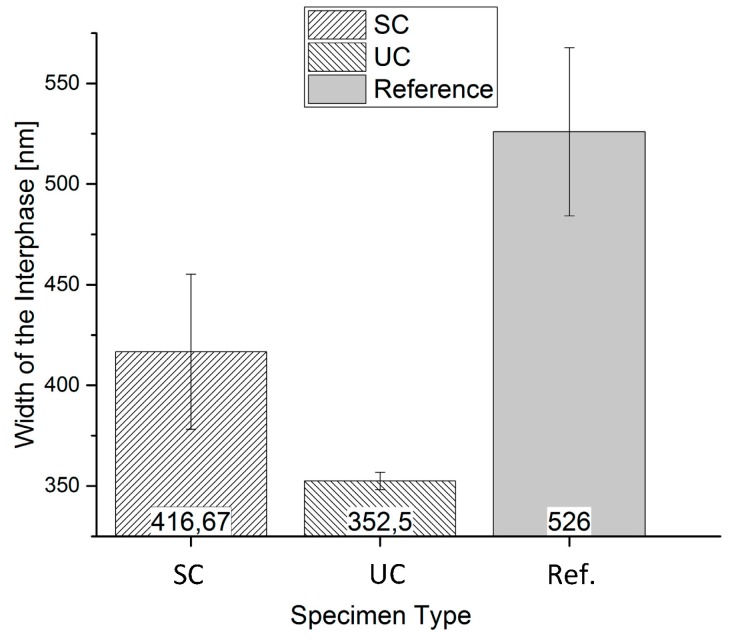
Interphase width for the influence of the fiber sizing measured with the introduced nano-scratch method with sized fibers (SC) and unsized fibers (UC).

**Figure 5:**
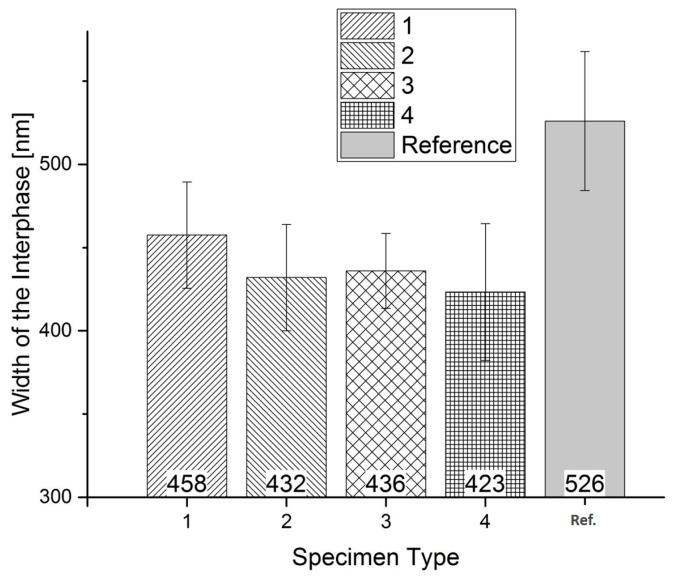
Interphase width for the influence of the processing parameters measured with the introduced nano-scratch method.

To determine the mean interphase width for each selected composite material, the nano-scratches are performed on different fibers within the cutting plane of the corresponding sample. Subsequent to the nano-scratch, an AFM image is taken of the sample surface as presented in Figure 2b,c. Table 10 lists the parameters used for the nano-scratch tests. The results of the nano-scratches for different sample types are presented in Figure 4 and Figure 5.

Figure 4 shows the results of the nano-scratch tests of the various samples to investigate the influence of the fiber sizing on the interphase formation. At least five scratches are performed on different glass fibers for each sample type, respectively. Depending on the glass fiber sizing, the width of the interphase varies widely. The specimens containing unsized glass fibers “UC” show the smallest interphase area and standard deviation with a value of 353 ± 4 nm. The interphase width for the sized glass fiber samples has a wider interphase with the mean values of 417 ± 39 nm. The reference specimens show an interphase width of 526 ± 42 nm.

It is noticeable that the values for the standard deviations vary significantly concerning the measured interphase width. The sample type containing unsized fibers has an extremely low standard deviation, whereas the specimen type with sized fibers, as well as the reference samples show higher standard deviations.

A possible reason represents the applied sizing on the glass fibers, which is also measured in the nano-scratch test and included to the interphase width. For this purpose, the quantity of the applied sizing of 0.95 mass percent according to the product data sheet is converted to a sizing thickness of 66.4 nm. On this occasion, a constant width of the applied sizing around the fiber is assumed. The calculated sizing thickness corresponds in a good approximation to the difference of the mean width of the interphase for a comparison of the unsized sample type (353 nm) and the sized sample type (417 nm).

The wider interphase area of the reference sample can be explained by unknown additives added to the industrially-manufactured material of the reference specimens. These additives usually improve the flowability and the dispersibility. In addition, the adhesion between glass fiber sizing and the matrix material can be optimized by additives.

The results of the nano-scratching tests for the specimens influenced by the processing parameters of the injection molding are shown in Figure 5. The interphase widths are measured for every specimen type. At least five scratches are performed on different glass fibers for each sample type, respectively.

The reference specimens show the widest interphase area of 526 ± 42 nm. Specimen Numbers 2 and 3 are closely matched at a width of 432 ± 32 nm and 436 ± 22 nm. The smallest width can be measured at Specimen Number 4 with 423 ± 41 nm. Differences of approximately 100 nm in the interphase widths are measured for the same material. To identify the injection molding parameter with the highest influence on the interphase formation, the experimental design has to be evaluated. The main effects plot for the influences on the interphase is shown in Figures 14–16.

The investigations in the present study mentioned above are performed with a semi-crystalline thermoplastic matrix material. In a semi-crystalline thermoplastic material, a crystallization occurs in a privileged place at the interfaces of the fibers, and the degree of crystallization depends on the process conditions of the crystallization. Amorphous thermoplastics are not able to crystallize because of their macromolecular structure, and correspondingly, it is not possible to form a long-range order like a semi-crystalline structure. In amorphous thermoplastics, there is only a near-range order. On this basis, it is obvious to study the possibility of the formation and the detection of an interphase in amorphous fiber-reinforced thermoplastics.

**Figure 6:**
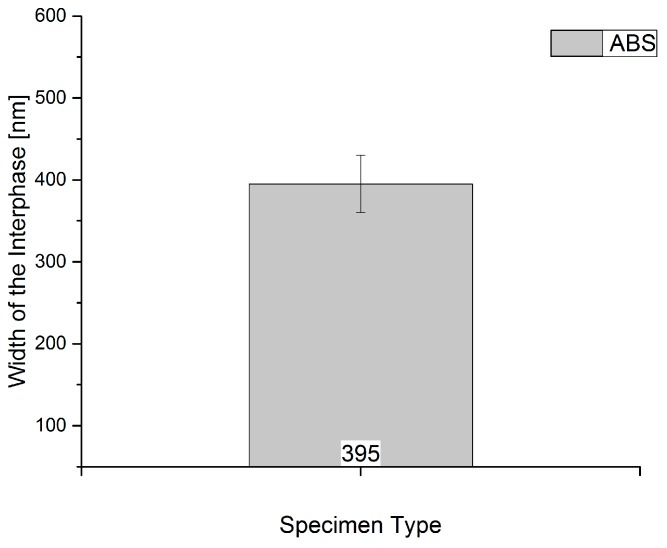
Interphase width for the influence of the amorphous material measured with the introduced nano-scratch method.

Figure 6 lists the values for the width of the interphases in amorphous ABS-GF20 samples. It can be seen that an interphase is detectable by using the applied nano-scratch method. The interphase width amounts 395 ± 35 nm for the amorphous material.

Despite the amorphous structure of the ABS matrix, the formation of an interphase occurs between the matrix and the glass fiber or rather the glass fiber sizing. The absolute values of this interphase width deviate compared to the values of the semi-crystalline samples. Since the structure of the macromolecules from the ABS differs from the macromolecular chain structure of the PBT material, a comparison of the overall width of the interphase is not appropriate. The formation of the interphase in an amorphous thermoplastic shows that the interphase is not only a phenomenon of crystallization. Similar to semi-crystalline thermoplastics, the amorphous thermoplastics form a three-dimensional interphase due to a thermodynamically-controlled interdiffusion of the macromolecules and the molecules of the glass fiber sizing [8].

## 5. Determination of the Mechanical Properties

**Figure 7:**
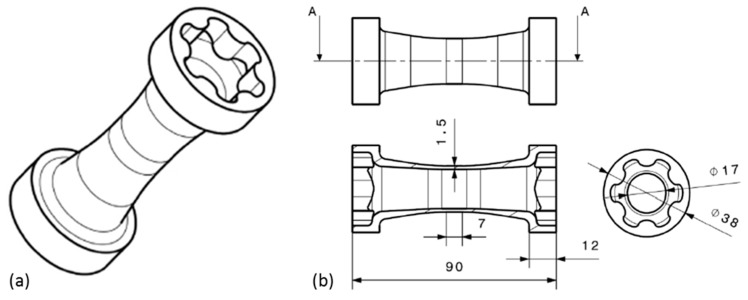
Tube specimen geometry for experimental investigations: (**a**) 3D representation; (**b**) connecting dimensions [36].

In the present contribution, the characterization of the macroscopic mechanical properties of the presented short glass fiber-reinforced thermoplastics is performed by means of tensile tests. Therefore, tube specimens introduced by Kaiser et al. [36] are used. Figure 7 shows the tube specimen geometry with a 3D representation of the specimen in (a) and the connecting dimensions in (b). Besides the possible application of multi-axial loading cases, the main feature of the specimen is a unidirectional fiber orientation in the central measurement section. The fiber orientation in the measurement section is determined by Kaiser with µCT measurements and micro sections [36]. The mechanical testing of the tube specimens is performed by uniaxial tensile tests to determine the mechanical properties in the fiber direction of the specimens and by internal pressure tests to determine the mechanical properties perpendicular to the fiber direction.

To convert the measured loads of the tensile tests into the required true strains for a stress-strain diagram, the following formula is used [37]. In Formula (1), the true strain $\sigma_{\mathrm{true}}$ in MPa is calculated with the load F in N applied to the specimen and the effective cross-sectional area $A_{a}$ in mm^2^ of the specimen.

(1) $$\sigma_{\mathrm{true}}=\frac{F}{A_{a}}$$

While the load is applied to the specimen, the wall thickness of the tube specimen is reduced due to the necking of the specimen, and therefore, the effective cross-sectional area decreases. To measure the wall thickness during the tests, a magnetostatic sensor is used. The effective cross-sectional area is calculated using Formulae (2)–(7).

(2) $$A_{a}=\frac{\pi }{4}\cdot \left({\bar{D}}^{2}-{\bar{d}}^{2}\right)$$

with:

(3) $$\bar{d}=\bar{D}-2\bar{s}$$

and:

(4) $$\bar{D}=\bar{D_{0}}\cdot \left(1+{\bar{\varepsilon }}_{t}\right)$$

and:

(5) $$\bar{s}={\bar{s}}_{0}\left(1+{\bar{\varepsilon }}_{r}\right)$$

⇔

(6) $${\bar{\varepsilon }}_{r}=\frac{\bar{s}}{\bar{s_{0}}}-1$$

(7) $$A_{a}=\pi \cdot \left(\bar{D_{0}}\cdot {\bar{s}}_{0}\cdot \left(1+{\bar{\varepsilon }}_{t}\right)\cdot \left(1+{\bar{\varepsilon }}_{r}\right)-{{\bar{s}}_{0}}^{2}\cdot {\left(1+{\bar{\varepsilon }}_{r}\right)}^{2}\right)$$

In Formulae (2)–(7), the following symbols are used:

- $\bar{D_{0}}$: average outer diameter of the tube specimen before the test started measured four times around the tube specimen with a caliper in mm;
- $\bar{D}$: current outer diameter during the test in mm;
- $\bar{d}$: current internal diameter during the test in mm;
- ${\bar{s}}_{0}$: wall thickness of a representative measurement point before the test started in mm;
- $\bar{s}$: current wall thickness during the test in mm;
- ${\bar{\varepsilon }}_{t}$: tangential strain (**-**);
- ${\bar{\varepsilon }}_{r}$: radial strain (-).

**Figure 8:**
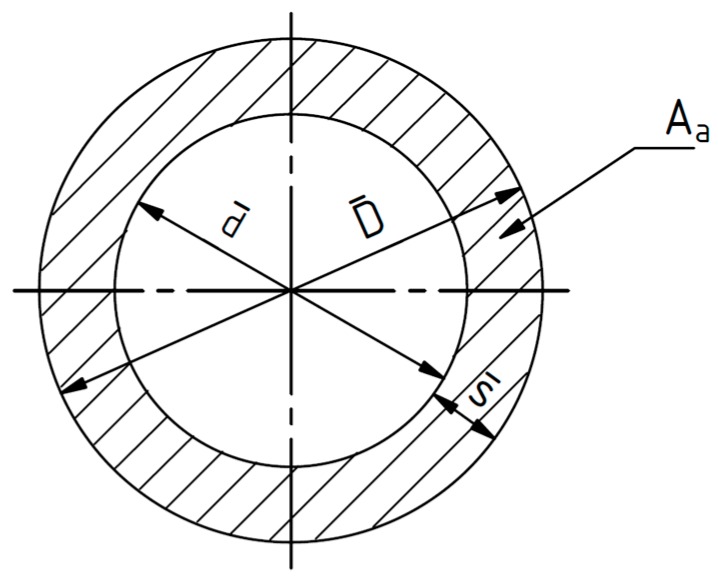
Geometric variables to calculate the effective cross-sectional area of the tube specimens.

The symbols used in Formulae (2)–(7) are displayed in Figure 8. The mechanical material properties perpendicular to the fiber orientation are measured by means of internal pressure tests. This load case is realized by a constant flow of a fluid into the sealed, parallel test area of the tube test specimen. The flow rate is set to 2 cm^3^/min, which refers to a strain rate of 1.3%/min. Analogous to the calculation of the true stresses of the tensile tests, the true stresses of the internal pressure tests are calculated using Formulae (8) and (9). The pressure p is measured in bar.

(8) $$\sigma_{\mathrm{true},\mathrm{internal}}=\frac{F}{A_{a}}$$

(9) $$\sigma_{\mathrm{true},\mathrm{internal}}=\frac{p\cdot \bar{\left({\bar{D}}_{0}\cdot \left(1+{\bar{\varepsilon }}_{t}\right)-2\cdot {\bar{s}}_{0}\cdot \left(1+{\bar{\varepsilon }}_{r}\right)\right)}}{2\cdot {\bar{s}}_{0}\cdot \left(1+{\bar{\varepsilon }}_{r}\right)}$$

**Figure 9:**
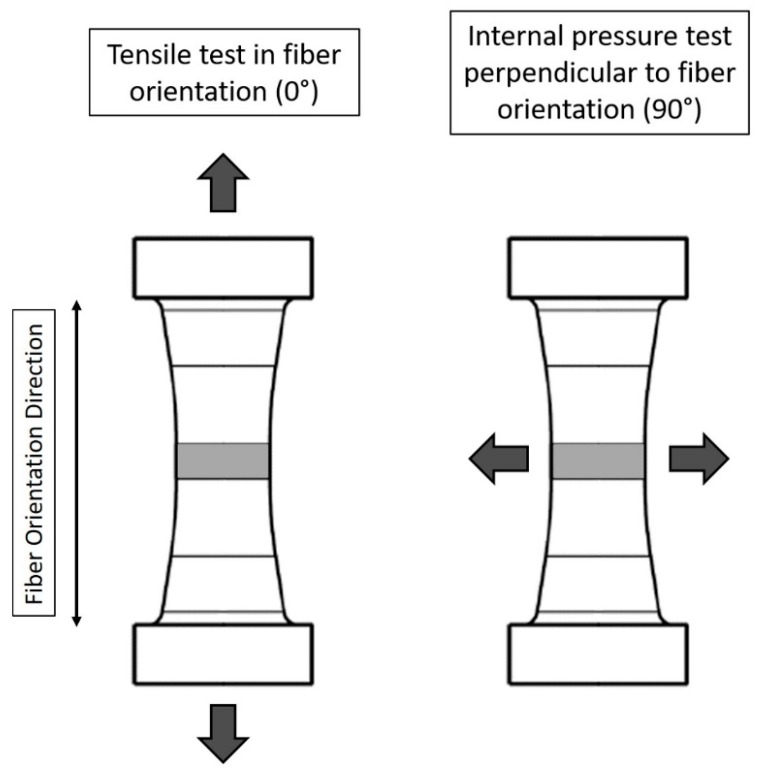
Representation of the performed tensile and internal pressure load cases in 0° and 90° fiber orientation of the tube specimen.

Figure 9 represents the performed tensile and internal pressure load cases in and perpendicular to the fiber orientation of the tube samples.

It is well known that the mechanical properties, especially the stiffness, of a composite are influenced by its fiber content. To verify the glass fiber content of the compounded granulate types in the study, matrix incinerations according to DIN EN ISO 1172 and DIN EN ISO 3451 are implemented. The results of the matrix incinerations are given in Table 12. The fiber weight fractions for the two granulate types are averaged for three measurements in each case. For the incineration, a temperature of 625 °C for at least 3 h is applied to incinerate all organic substances.

**Figure 10:**
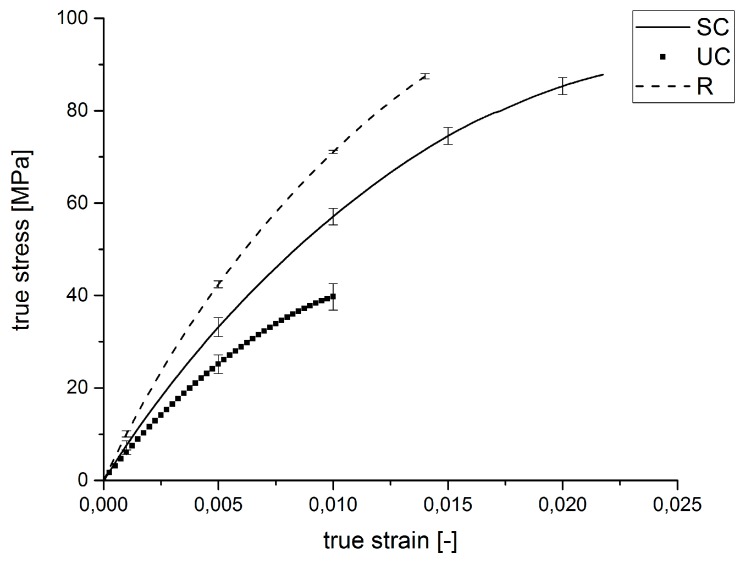
Average stress-strain diagram for the tensile tests of samples with (SC) and without (UC) glass fiber sizing and reference samples (R).

**Figure 11:**
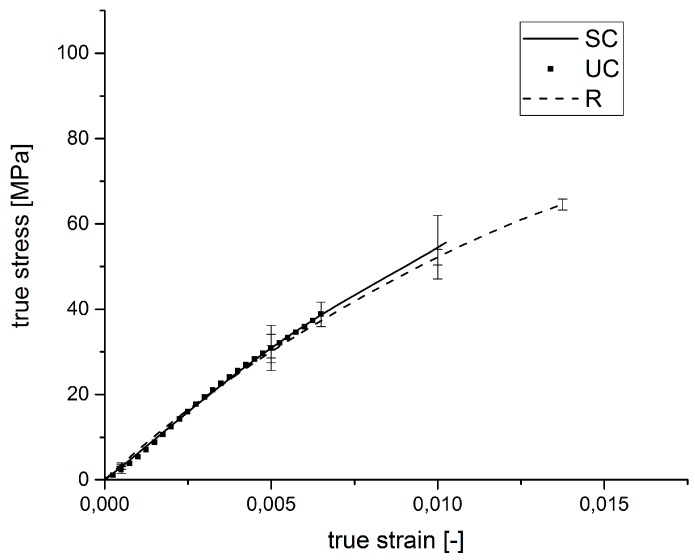
Average stress-strain diagram of the internal pressure tests for the samples influenced by the injection molding parameters.

The average glass fiber contents of the granulate types deviate slightly from the aspired 20 weight percent of the reference sample material. The influence of the glass fiber sizing on the mechanical properties is tested by at least ten tensile tests and ten internal pressure tests per specimen type. The applied parameters of the tensile testing are given in Table 11. The results are shown in Table 13 and Table 14, as well as in Figure 10 and Figure 11.

The results of the tensile tests are given by Figure 10 and Table 13. It is recognizable that the stress-strain behavior is influenced by the presence of a glass fiber sizing. In additional, differences in the stiffness of the sample types are determined for the specimen types.

The tensile tests with loads in the fiber direction of the tube specimens show that the tensile strength and the stiffness of the specimens with unsized glass fibers are lower than the tensile strength and stiffness of the samples with sized glass fibers, although the fiber content of the samples with unsized glass fibers is higher. In comparison, the stress-strain plot of the tensile tests from the reference specimens and the samples with the sized glass fibers show similar tensile strengths and stiffnesses, but the elongation at break is higher for the samples with sized glass fibers. Considering these results, it is evident that the glass fiber sizing has an enormous impact on the fiber-matrix adhesion, because the samples with unsized glass fibers break at significantly lower loads. The load cannot be transferred through the fibers because of a low adhesion between the matrix material and the glass fibers resulting from the absent sizing.

The results of the internal pressure tests for the samples influenced by the glass fiber sizing, as well as the reference specimens are listed in Table 14 and Figure 11.

The determined stress-strain diagrams of the internal pressure tests with loads perpendicular to the fiber direction of the manufactured tube specimens show similar stiffnesses for every sample type. The similarity of the stiffnesses results due to the reduced influence of the fibers on the mechanical composite properties when loads are applied perpendicular to the fiber orientation. In the internal pressure test, the fibers are oriented perpendicular to the applied load, and the load cannot be transferred through the fibers. In this load case, the fibers only act as inactive fillers, and therefore, only the matrix stiffness is measured. The different sample types show significant differences in the breaking strengths.

**Figure 12:**
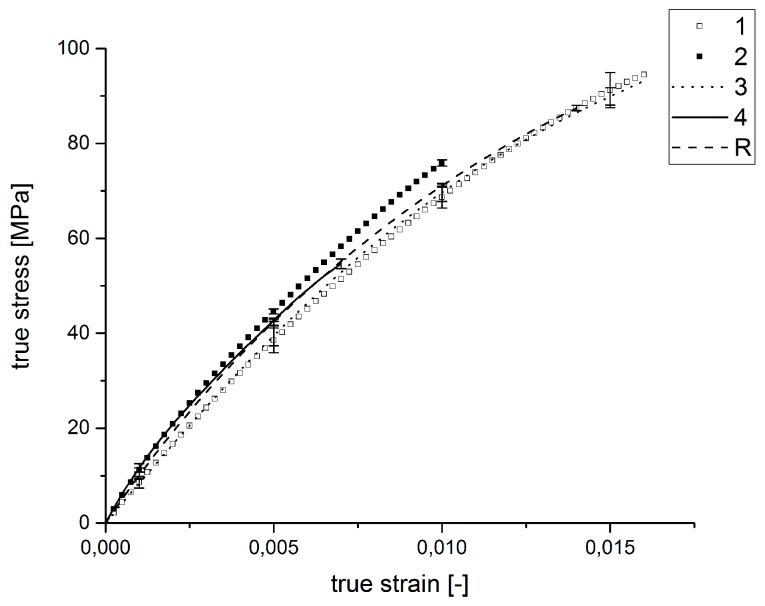
Average stress-strain diagram of the tensile tests for the samples influenced by the injection molding parameters.

Table 15 shows the results of the mechanical testing in the 0° direction to the fiber orientation of the tube specimens influenced by the injection molding parameters in the experimental design. The parameters for the applied tests are listed in Table 11. Connected to the values of Table 15, the corresponding stress-strain diagram follows in Figure 12.

It can be determined that tensile strengths and the elongations at break of the specimens differ significantly. The Specimen Types 1 and 3 exhibit higher tensile strengths at higher elongations at break than the other specimens. The mechanical properties can be compared with the reference specimens. Specimen Numbers 2 and 4 fail at lower tensile strengths and smaller strains. However, it can also be recognized that there are only small differences in the stiffnesses of the specimen types. Obviously, the stiffness of the samples is not influenced by the injection molding parameters.

**Figure 13:**
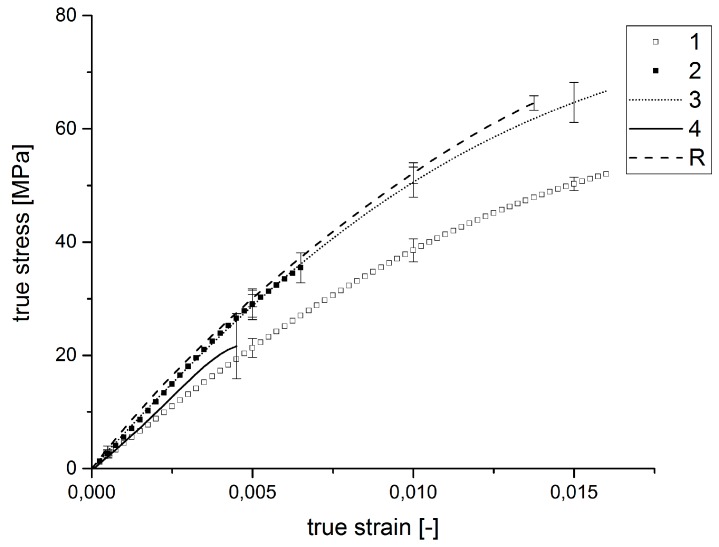
Average stress-strain diagram of the internal pressure tests for the samples influenced by the injection molding parameters.

Table 16 lists the average tensile strengths and elongations at break of the stress-strain behavior from the internal pressure tests conducted for the design of experiments. The corresponding stress-strain curves are shown in Figure 13.

Figure 13 and Table 16 indicate that Specimen Type 3 shows the highest average tensile strength with 66.7 ± 3.5 MPa for the internal pressure tests. The reference specimens have an average tensile strength of 64.5 ± 1.3 MPa. The elongations at break of Specimen Type 3 amount to 0.016, whereas the elongation of the references amounts to 0.013. Specimen Types 2 and 4 show significantly lower tensile strengths and elongations at break than the residual specimen types. While the stiffnesses of Specimen 2 and 3 and the reference sample are comparable, Samples 4 and 1 show lower stiffnesses. Hence, it can be concluded that there are significant differences in the fracture behavior of the various specimen types. The injection molding parameters influence the tensile strengths and the elongations at break of the samples appreciably, while the elastic moduli of the samples remain comparable.

## 6. Correlation of the Interphase Properties with the Mechanical Behavior

**Figure 14:**
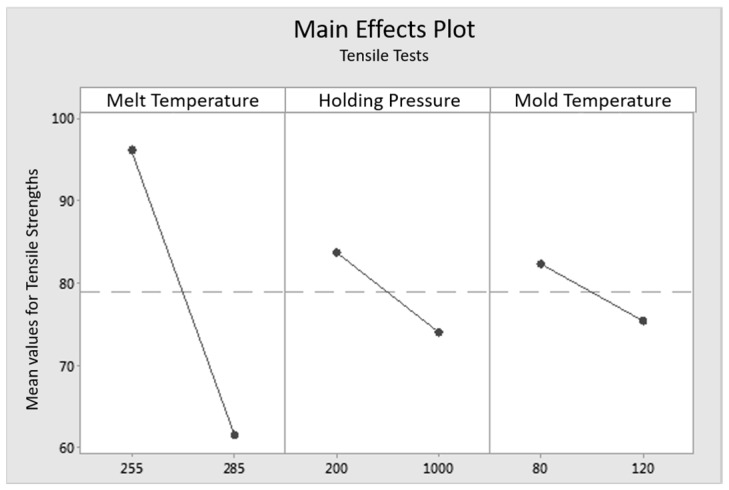
Main effects plot of tensile tests for the influencing factors “mass temperature”, “holding pressure” and “mold temperature” on the tensile load in the fiber orientation.

**Figure 15:**
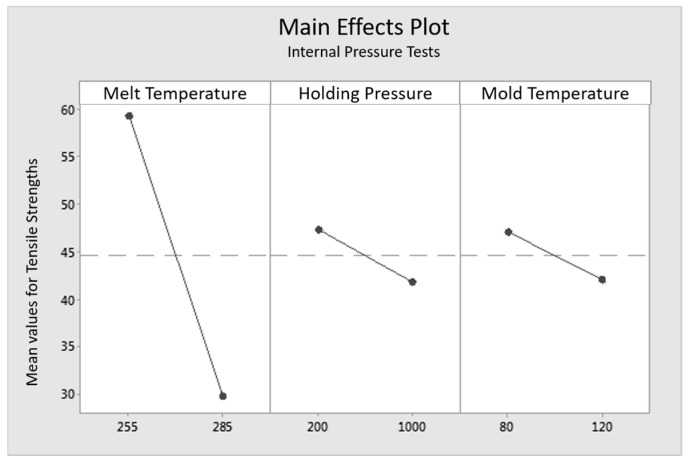
Main effects plot of tensile tests for the influencing factors “mass temperature”, “holding pressure” and “mold temperature” on the tensile load perpendicular to the fiber orientation.

**Figure 16:**
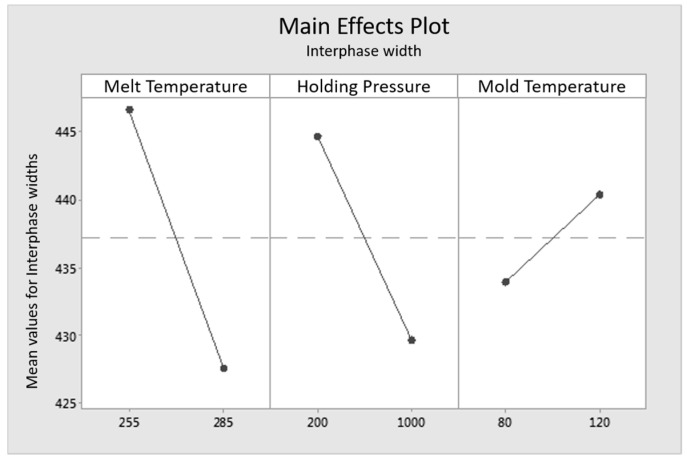
Main effects plot for the width of the overall interphase for the influencing factors “mass temperature”, “holding pressure” and “mold temperature”.

To correlate the formation of the interphase with the mechanical properties, an evaluation of the experimental design is implemented by MiniTab 17, a statistical evaluation program. The correlation of the measured widths of the interphases and the tensile strength of the load in 0° to the fiber orientation (tensile tests) and 90° to the fiber orientation (internal pressure tests) are shown in three main effects plots in Figure 14, Figure 15 and Figure 16. Main effects plots quantify the impacts of the respective factors to a quality feature (here: “tensile strengths” in 0° to the fiber orientation for the tensile tests, “tensile strengths” in 90° to the fiber orientation for the internal pressure tests and “width of the interphase” in the high and the low level setting). In a main effects plot, the gradient of the line describes the influence of the factor on the experimental results. The larger the gradient of the line, the higher the influence on the considered factor.

Figure 14 shows the main effects plot for the influencing factors temperature, holding pressure and mold temperature on the tensile load in 0° to the fiber orientation. The mass temperature of the melt has the highest effect on the tensile strength, while the factors holding pressure and mold temperature show less impact. Additionally, the lower level settings are connected with the higher tensile strengths.

Figure 15 shows the main effect plot for the tensile load perpendicular to the fiber orientation impacted by the internal pressure tests. Once again, the mass temperature of the melt shows the biggest impact on the mechanical properties of the specimens, whereas the effects of the holding pressure and the mold temperature are comparatively low. The respectively lower level settings for the factors relate to the higher tensile strengths.

The main effects plot pictured in Figure 16 shows that the mass temperature has the biggest effect on the width of the interphase. Additional, a major impact of the mass temperature can be seen. For both factors, the widest interphase is measured for the low level settings. The mold temperature can be detected as the smallest influence on the system. Concerning Figure 16, the parameter settings of the mass temperature and the holding pressure both have an impact on the interphase. At the lower mass temperature or rather the lower holding pressure, a wider interphase is expected. Conversely, a smaller interphase is formed by a higher mold temperature. This effect can be explained by a higher cooling rate of the melt [16]. The three factors “mass temperature”, “holding pressure” and “mold temperature” influence the width of the interphase by affecting the crystallization kinetics of the matrix material. The highest impact on the mechanical properties and also on the interphase is given by the mass temperature. The experimental design shows a clear dependency between the tested factors and the resulting effects in the main effects plots. However, the interaction between the factors themselves cannot be dissolved because of the experimental design type. Further tests to complete the fractional factorial design to a full factorial design have to be implemented to recognize the interactions between the factors.

Regarding the stress-strain curves of the mechanical testing and the determination of the interphase width, a correlation between interphase width and material strength can be stated. The study to determine the influence of the injection molding settings shows that the settings influence the formation of the fiber-matrix-interphase and therefore have an impact on the tensile strength, as well as the elongation at break. The width of the interphase does not affect the stiffness, but the fracture behavior, including tensile strength and elongation at break. Comparing the results of the nano-scratch tests in Figure 5 and the tensile testing in Figure 12, the tensile strength and elongations at fracture correlate with the width of the interphase. The widest interphases and breaking strengths are measured for the reference specimen and Specimen Type 1, and furthermore, the smallest interphase and the lowest breaking strengths are detected for Specimen Types 2 and 4.

## 7. Summary and Conclusions

The present study is focused on the influence of the processing parameters on the interphase formation in short fiber-reinforced thermoplastics. For this purpose, different experimental methods are applied to determine the mechanical properties of the composites regarding the existence of a fiber-matrix interphase.

To characterize the macroscopic mechanical properties, tensile tests are carried out in and perpendicular to the fiber orientation of the injection molded test specimen. The microstructural study of the interphase is done by means of a nano-tribological indentation technique according to the methodology of Schöneich et al. [4]. In this method, a cube corner indenter tip of a nano-indenter is impressed into the thermoplastic matrix and moved up to the glass fiber at a constant depth. The progression of the controlled normal force to maintain the constant scratch-depth is referred to an interphase between the matrix and the fiber. The knowledge gained from the study is summarized below:

The fiber sizing affects the fiber-matrix adhesion and the fracture properties due to the interphase formation.

(Hypothesis 1)

Sized and unsized glass fibers are compounded into a matrix material to determine the influence of the glass fiber sizing on the fiber-matrix adhesion and samples that are injection molded. Additionally, equivalent industrial-grade composite samples are injection molded using the same injection molding parameters. The samples are tested in fiber orientation direction (tensile tests) and perpendicular to the fiber orientation direction (internal pressure tests), and the width of the interphase is examined by means of nano-scratching.

It can be shown that samples with unsized glass fibers have lower tensile strengths and significantly smaller interphases than the samples with coated glass fibers. Furthermore, the nano-scratch results display an additional thickness around 60 nm, which is induced by the sizing of glass fibers. The results show that the fiber sizing has a huge impact on the fiber-matrix adhesion and therefore on the breaking strength.

The formation and the width of the interphase are influenced by the processing parameters of the injection molding process by crystallization kinetics.

(Hypothesis 2)

The evaluation of the experimental results in the DoE study reveals that the melt temperature has the highest influence on the tensile strength in the 0° and 90° fiber orientation, as well as on the interphase width. As a consequence, the highest tensile strengths and widest interphases are identified at a lower melt temperature. The holding pressure and the mold temperature have a smaller effect on the tensile strength and the interphase width. Regarding the holding pressure, higher strengths and interphase widths are measured at the lower factor level. At a higher mold temperature, a wider interphase is measured, but a slight reduction of the tensile strengths of the investigated samples is also detected. However, the performed DoE study does not allow further conclusions about interactions and commingling of the individual process parameters, since they are not explicitly resolved in screening test plan.

The interphase is not only a crystallization phenomenon for semi-crystalline thermoplastics.

(Hypothesis 3)

In semi-crystalline thermoplastic composites, the presence of an interphase can be explained by concentrated nucleation sites and increased spherulitic formation in the vicinity of the fiber material. Amorphous thermoplastics, however, do not contain crystalline structures due to their near order polymer chain configuration. In this work, several samples of short glass fiber-reinforced amorphous thermoplastics (ABS-GF20) are produced and tested for their interphase via nano-scratching. As a result of the present contribution, fiber-matrix interphases can be identified in amorphous polymer composites, as well. Therefore, the interphase cannot solely be described as a crystallization phenomenon. In the formation process of the interphase, additional thermodynamic aspects, such as the interdiffusion of the matrix macromolecules, as well as the interaction with the fiber coating, are of challenging importance and should be studied further.

The width of the interphase correlates with the mechanical behavior of thermoplastics.

(Hypothesis 4)

The results of the performed tensile tests, as well as the nano-scratches produced with different injection molding parameters show that the width of the interphase can be influenced by the parameters “melt temperature”, “holding temperature” and “mold temperature”. The resulting interphase widths and tensile strengths, as well as the elongations at break for the processed samples vary significantly while the stiffnesses remain comparable in the mechanical tests. A correlation of the interphase thickness with the breaking strength and the elongation at break can be seen. The interphase is an important area for the adhesion between the matrix material and the fibers. In further studies, the influence of the interphase on the mechanical long time behavior will be studied in fatigue tests.

## Figures and Tables

**Table 1:** Material properties of the investigated polybutylene terephthalate reinforced with 20 wt % of short glass fibers (PBT GF20) “Pocan B 3225/Lanxess” [28].

Property	Value	Unit
Density	1460	kg/m^3^
Tensile modulus	7100	MPa
Tensile stress at break	120	MPa
Tensile strain at break	3.4	%
Melting temperature	225	°C

**Table 2:** Material properties of the investigated ABS-GF20 “Polyman FABS-20-GF/A. Schulman” [29].

Property	Value	Unit
Density	1200	kg/m^3^
Tensile modulus	5500	MPa
Tensile stress at break	65	MPa
Tensile strain at break	2	%
Melting temperature	105	°C

**Table 3:** Material properties of the investigated PBT matrix material “Pocan^®^ B 1305/Lanxess” [30].

Property	Value	Unit
Density	1310	kg/m^3^
Tensile modulus	2800	MPa
Tensile stress at break	60	MPa
Tensile strain at break	9.0	%
Melting temperature	225	°C

**Table 4:** Material properties of the investigated glass fibers (unsized [31]/sized [32]).

Property	Value	Unit
Sized fibers (GF CS 7968/Lanxess)		
Glass type	E-glass	-
Fiber sizing	Silane-based polymer-coating	-
Sizing content	approximately 0.9	wt %
Mean fiber length	4.5	mm
Mean fiber diameter	11	µm
Unsized fibers (GF MF 7980/Lanxess)		
Glass type	E-glass	-
Fiber sizing	-	-
Sizing content	-	wt %
Mean fiber length	190	µm
Mean fiber diameter	14	µm

**Table 5:** Applied parameters of the compounding process.

Parameter	Setting	Unit
Temperature of the heating zones (1–10)	280 in all heating zones	°C
Drive	100	1/min
Output feeder matrix	1	kg/h
Output feeder fibers	0.25	kg/h
Pellet length	1.5	mm

**Table 6:** Listing of the utilized composites for the specimen processing to examine the influencing factors on the formation of a fiber-matrix interphase.

Influencing Factor	Material	Trading Name	Specimen Shortcut
Reference	PBT-GF20	Pocan^®^ B 3225	Reference
Process of injection molding	PBT-GF20	Pocan^®^ B 3225	DoE 1–4
With glass fiber sizing	PBT-GF20	Pocan^®^ B1305 + GF CS 7968	BC
Without glass fiber sizing	PBT-GF20	Pocan^®^ B1305 + GF MF 7980	UC
Morphology of the matrix	ABS-GF20	POLYMAN FABSABS 20 GF	ABS

**Table 7:** Constant adjusted processing parameters to process the specimen in the injection molding process.

Parameter	Setting	Unit
Dosage	76.5	(cm^3^)
Circumferential speed	5	(m/min)
Injection pressure	1600	(bar)
Volume flow	30	(cm^3^/s)
Switchover volume	2.3	(cm^3^)
Holding pressure time	2	(s)
Residual cooling time	36	(s)
Desiccation of the granulate before processing	120/2 (PBT-GF20)	(°C/h)
	80/3 (ABS-GF20)	
Residual moisture of the granulates	0.001	(%)
Barrel temperature	270 (PBT-GF20)	(°C)
	250 (ABS-GF20)	
Mold temperature	100 (PBT-GF20)	(°C)
	60 (ABS-GF20)	
Holding pressure	600 (PBT-GF20)	(bar)
	600 (ABS-GF20)	

**Table 8:** Level settings for the low, the reference and the high level setting.

Level Setting	Melt Temperature (°C)	Holding Pressure (bar)	Mold Temperature (°C)
−1	255	200	80
Reference Setting	270	600	100
+1	285	1000	120

**Table 9:** Applied fractional factorial experimental design.

Level Setting	Melt Temperature (°C)	Holding Pressure (bar)	Mold Temperature (°C)
1	255	200	120
2	285	200	80
3	255	1000	80
4	285	1000	120
Reference Setting	270	600	100

**Table 10:** Applied parameters of the nano-scratching tests.

Parameter	Setting	Unit
Indenter tip	Cube corner	-
Scratches per sample	5 on different glass fibers	-
Scratch depth pre scratches	100	nm
Scratch depth main scratch	150	nm
Scratch velocity	0.1	µm/s
Path length per scratch	10	µm

**Table 11:** Applied parameters of the tensile tests and the internal pressure test.

Parameter	Setting	Unit
Strain Rate Tensile Testing	1	mm/min
Flow Rate for Internal Pressure Test	2	cm^3^/min
Corresponding Strain Rate for Internal Pressure Test	1.3	%/min

**Table 12:** Results of the incineration of the matrix material according to DIN EN ISO 1172 and DIN EN ISO 3451.

Granulate Type	Averaged Glass Fiber Content (wt %)
SC	16.49 ± 0.52
UC	17.92 ± 1.32

**Table 13:** Averaged tensile strengths and elongations at break of the tensile tests of samples with (SC) and without (UC) glass fiber sizing and reference samples.

Specimen Name	Tensile Strength (MPa)	Elongation at Break (-)
UC	39.7 ± 2.9	0.010 ± 0.002
SC	87.8 ± 1.9	0.021 ± 0.003
Reference	87.4 ± 0.6	0.014 ± 0.001

**Table 14:** Average tensile strengths and elongations at break of the internal pressure tests of samples with (SC) and without (UC) glass fiber sizing and reference samples.

Specimen Name	Tensile Strength (MPa)	Elongation at Break (-)
UC	38.8 ± 2.8	0.007 ± 0.001
SC	55.7 ± 7.4	0.011 ± 0.002
Reference	64.6 ± 1.3	0.014 ± 0.002

**Table 15:** Average tensile strength and elongation at break of the stress-strain behavior from the tensile tests for the samples influenced by the injection molding parameters.

Specimen Name	Tensile Strength (MPa)	Elongation at Break (-)
1	95.5 ± 5.3	0.016 ± 0.003
2	75.9 ± 0.6	0.010 ± 0.002
3	93.1 ± 2.5	0.016 ± 0.002
4	54.6 ± 1.0	0.007 ± 0.003
Reference	87.4 ± 0.6	0.014 ± 0.001

**Table 16:** Averaged tensile strength and elongation at break of the stress-strain behavior of the internal pressure tests for the samples influenced by the injection molding parameters.

Specimen Name	Tensile Strength (MPa)	Elongation at Break (-)
1	52.0 ± 1.2	0.016 ± 0.002
2	35.4 ± 2.6	0.007 ± 0.003
3	66.7 ± 3.5	0.016 ± 0.006
4	21.6 ± 5.7	0.005 ± 0.002
Reference	64.6 ± 1.3	0.014 ± 0.005
